# Pulmonary hypertension associated with rheumatoid arthritis: A case report and causal association verification through Mendelian randomization

**DOI:** 10.1097/MD.0000000000043116

**Published:** 2025-07-25

**Authors:** Jingwei Liu, Chunyan Rong, Yin Wang, Baoguo Wang, Xuhan Liu, Weihua Zhang

**Affiliations:** a Department of Cardiovascular Medicine, The First Hospital of Jilin University, Changchun, Jilin, China.

**Keywords:** autoimmune disease, connective tissue disease, Mendelian randomization analysis, pulmonary hypertension, rheumatoid arthritis

## Abstract

**Rationale:**

Rheumatoid arthritis (RA) is a chronic systemic autoimmune connective tissue disease characterized by joint swelling and pain, affecting multiple organs. Pulmonary hypertension (PH) is a known but relatively rare pulmonary vascular complication in RA patients. The pathophysiological mechanisms linking RA and PH are diverse, and Mendelian randomization analysis can be used to explore their correlation.

**Patient concerns:**

A 43-year-old woman presented with swelling and pain in multiple joints of both hands and dyspnea on exertion lasting over 6 months.

**Diagnoses:**

Laboratory tests showed elevated rheumatoid factor, erythrocyte sedimentation rate, C-reactive protein, and anticyclic citrullinated peptide antibody. Echocardiogram revealed PH and mild tricuspid regurgitation. Pulmonary computed tomography scan showed no signs of interstitial lung disease. Pulmonary function tests indicated severe reduction in diffusion capacity and alveolar volume. Right heart catheterization confirmed postcapillary PH with elevated mean pulmonary artery pressure and pulmonary vascular resistance.

**Interventions:**

The patient received antirheumatic drugs combined with PH-targeted therapies for 4 months.

**Outcomes:**

At follow-up, the patient’s pulmonary artery pressure returned to normal levels.

**Lessons:**

The pathophysiology of RA-associated PH involves multiple inflammatory components. Accurate diagnosis requires right heart catheterization, pulmonary computed tomography, and pulmonary function testing. Combined antirheumatic and PH-targeted treatments can effectively reduce autoantibody levels and alleviate PH.

## 1. Introduction

Rheumatoid arthritis (RA) is a chronic systemic autoimmune joint disease, whose main characteristics include destruction of cartilage and bone, synovial hyperplasia, chronic inflammation, and pannus formation.^[1]^ The most common symptoms for RA are joints’ swelling and pain and early morning joint stiffness. Prolonged progression of RA can lead to joint deformity, loss of motor function and eventually disability. The prevalence of RA is about 1% to 2%, which of women is 2 to 3 times higher than men.^[2]^ The pathogenesis of RA has not been elucidated, which may refer to multiple factors such as immune responses, inflammatory factors, and abnormal oxidative stress.^[1]^

As a connective tissue disease (CTD), RA can affect multiple organs throughout the body. RA has many extra-articular manifestations including pulmonary manifestations, which affect over 80% of RA patients.^[3]^ In about 20% of RA patients, pulmonary lesions may occur before articular symptoms and are often detected within 5 years. RA pulmonary manifestations are characterized by involvement of all pulmonary structures, which usually include interstitial pneumonia and fibrosis, rheumatoid nodules, organizing pneumonia, bronchiectasis, obliterated bronchiolitis, follicular bronchiolitis, and pleural effusion.^[4]^

Pulmonary hypertension (PH) is a well-known pulmonary vascular complication of CTDs, though it is rare in RA patients. The pathophysiological mechanisms of PH related to RA are diverse. Multiple pulmonary manifestations of RA can all contribute to chronic lung disease or hypoxia,^[5]^ contributing to Group 3 PH. On the other hand, pulmonary arterial hypertension (PAH) can occur secondary to various CTDs, due to autoimmune and inflammatory reactions causing extensive pulmonary vascular damage. However, the relationship between RA and PAH has not been established.^[6]^ Here, we present a case of an RA patient with PH whose pulmonary artery pressure normalized following treatment with PH-targeted and antirheumatic drugs. This case highlights the potential role of inflammation in RA-PH and suggests that combined treatment strategies targeting both pulmonary vascular remodeling and systemic inflammation may be effective.

Mendelian randomization (MR) analysis is an analytical method that explores the causal associations between exposure and outcome. We extract genetic variants of single-nucleotide polymorphisms (SNPs) as instrumental variables for an exposure factor, and combine them with the outcome factor through the least squared method. As genetic variants are not influenced by disease status, the reverse causation in observational studies can be avoided.^[7]^ We designed a 2-sample MR study to verify the causal relationship between RA and PAH. All data were obtained from publicly available GWASs in IEU open GWAS database (https://gwas.mrcieu.ac.uk/).

## 2. Case Report

A 43-year-old woman was admitted to the First Hospital of Jilin University with complaints of swelling and pain in multiple joints of both hands for 2 years and exertional dyspnea for 6 months. She has no notable medical history. On admission, her blood pressure and heart rate were 126/80 mm Hg and 113 bpm respectively. Physical examination did not reveal jugular venous distention. The coarse breath sounds were auscultated bilaterally, but no crackles were noted. Her heart exhibited a regular rhythm, with no additional heart sounds or cardiac murmurs, although an increased P2 was observed. Abdominal examination revealed a soft abdomen without tenderness or rebound tenderness, and the hepatojugular reflux sign was negative. There was no edema in either lower extremity. Swelling and tenderness of multiple joints was found in both hands.

Laboratory findings were shown in Table 1. Elevated levels of rheumatoid factor (RF), erythrocyte sedimentation rate (ESR), C-reactive protein (CRP), and anti-cyclic citrullinated peptide antibodies (ACPA) were notably observed. Additionally, positive results were obtained for the IgM type of anti-cardiolipin antibodies and homogeneous antinuclear antibodies (ANA). The arterial blood gas analysis indicated a decrease in oxygen partial pressure and arterial oxygen saturation. The hemoglobin was mildly elevated. B type natriuretic peptide (BNP) and cardiac troponin I (cTnI) were in the normal range. Renal function, coagulation function and thyroid function tests returned normal results. Additionally, the patient’s HIV antibody (HIV-Ab), hepatitis B surface antigen (HBsAg), hepatitis B core antibody (HBcAb), and hepatitis C antibody (HCV-Ab) tests were all negative.

**Table 1:** Laboratory test results at first visit and follow-up visit.

Project	First visit	1-mo follow-up	4-mo follow-up	Reference range
PaO_2_ (mm Hg)	56.4			83–108
PaCO_2_ (mm Hg)	30.4			35–48
SO_2_ (%)	86.7			95–98
WBC (×10^9^/L)	7.82	6.62	5.87	3.5–9.5
HB (g/L)	152	130	128	115–150
CRP (mg/L)	21.94	1.26	2.72	0–1
ESR (mm/h)	34		28	0–20
RF (IU/mL)	300.66	72.37	32.99	0–14
ASO (IU/mL)	55.48	51.81		0–200
ACPA (RU/mL)	>200	189	156	0–5
ACA IgM (MPL)	42			0–12
Homogeneous ANA	1:1000 (+)		1:1000 (+)	(−)
COVID-19	(−)			(−)
NT-pro-BNP (pg/mL)	85.1	35.1	29.9	0–125
cTnI (ng/mL)	<0.012	<0.012	<0.012	0–0.034
AST (U/L)	34.6	18.9	35.5	13.0–35.0
ALT (U/L)	45.2	11.5	34.5	7.0–40.0

ACA = anti-cardiolipin antibody, ACPA = anti-cyclic citrullinated peptide antibody, ALT = alanine aminotransferase, ASO = antistreptolysin O, AST = aspartate aminotransferase, CRP = C-reactive protein, ESR = erythrocyte sedimentation rate, HB = hemoglobin, RF = rheumatoid factor, WBC = white blood cell.

**Figure 1.**
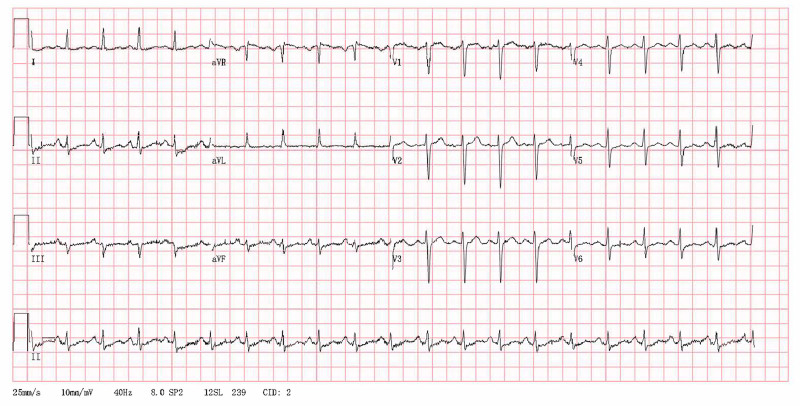
Patient’s electrocardiogram: heart rate: 120 bpm; P-R interval:162 (120–200) ms; QRS duration: 82 (80–100) ms; QT/QTC: 312/440 (320–400) ms; P-R-T: 61/7/49. Diagnosis: Sinus tachycardia. QRS = QRS complex, QT = QT interval, QTc = corrected QT interval.

**Figure 2.**
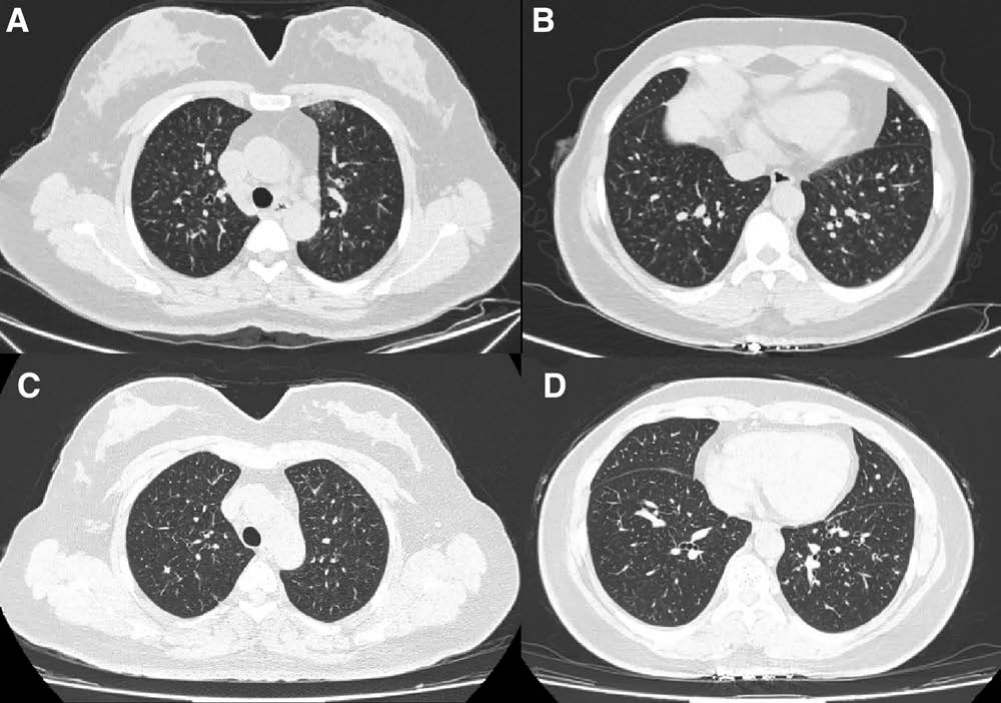
CT scan of the lungs: (A and B): At the initial visit: Bronchitis, double viral pneumonia and multiple inflammatory nodules in both lungs. (C and D): At the return visit: Remission of double pneumonia.

**Figure 3.**
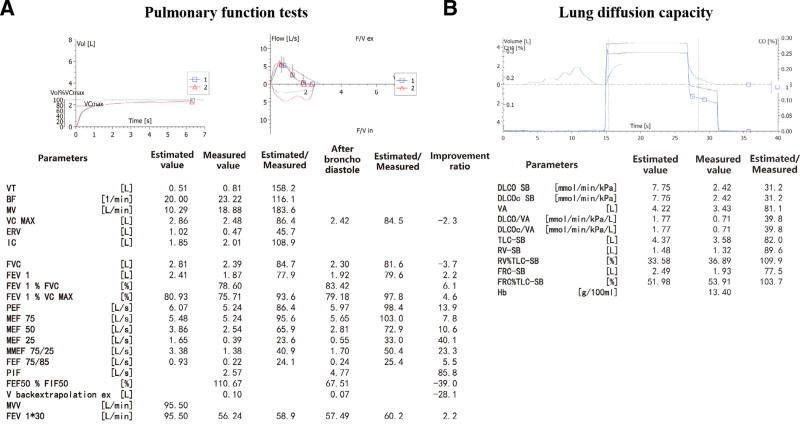
Pulmonary function tests: (A) Lung volumes and bronchodilation tests: Mild obstructive ventilation dysfunction. The bronchodilation tests were negative. (B): Lung diffusion capacity: Pulmonary dispersion function and alveolar diffusion volume decreased severely.

The electrocardiogram indicated sinus rhythm with a heart rate of 120 bpm (Fig. 1). The echocardiogram revealed a left ventricular ejection fraction of 69%, a right ventricular diameter of 20 mm, a right atrial diameter of 35 × 43 mm, a main pulmonary diameter of 27 mm (equal to the aortic root diameter of 27 mm), a tricuspid annular plane systolic excursion (TAPSE) of 20 mm. PH and mild tricuspid regurgitation were indicated by a tricuspid regurgitation velocity of 4.07 m/s and pressure gradient of 66 mm Hg (Table 2). Chest CT findings included bronchitis, bilateral pneumonia and multiple inflammatory nodules in both lungs. There were scattered patchy ground-glass opacities and linear high-density opacities in bilateral lungs (Fig. 2A and B). Abdominal CT scan indicated fatty liver and left renal stone. Pulmonary function tests demonstrated mild obstructive ventilation dysfunction, characterized by an FEV1 (forced expiratory volume in the first second) representing 77.9% of the estimated value, an FEV1/FVC (forced vital capacity) ratio of 78%, and a negative bronchodilation test (Fig. 3A). In contrast, the dispersion function exhibited a significant decrease, with lung diffusion capacity for carbon monoxide (DLCO) at 31% of the estimated value, a marked reduction in alveolar diffusion volume (VA), and a DLCO/VA ratio of 39% of the estimated value (Fig. 3B). The 6-minute walking distance (6MWD) was <165 meters.

**Table 2:** The echocardiogram results at first visit and follow-up visit.

Measured variables	First visit	4-mo follow-up
Left ventricular end-diastolic diameter (mm)	44	46
LVEF (%)	69	67
Right ventricular internal diameter (mm)	20	20
Right atrial diameter (mm)	35 × 43	–
Main pulmonary diameter (mm)	27	–
Aortic root diameter (mm)	27	26
TAPSE (mm)	20	–
TRV (m/s)	4.07	2.8
PG (mm Hg)	66	32

LVEF = left ventricular ejection fraction, PG = pressure gradient, TAPSE = tricuspid annular plane systolic excursion, TRV = tricuspid regurgitation velocity.

Right heart catheterization (RHC) indicated PAWP at 20 mm Hg, mPAP at 48 mm Hg, cardiac output at 4.3 L/min, cardiac index CI at 2.5 L/min/m^2^, and PVR at 6.51 Wood (Table 3). The diagnosis was combined postcapillary PH. In this case, the patient’s RAP was 18 mm Hg. The patient might have right heart failure or excessive volume load, etc. No signs of pulmonary artery thrombosis were observed during RHC.

**Table 3:** Right heart catheterization results.

Measured variables	Value
Right atrial pressure, mean (RAP)	20/15 (18)
Pulmonary artery pressure, mean (mPAP)	65/38 (48) mm Hg
Pulmonary arterial wedge pressure, mean (PAWP)	24/15 (20) mm Hg
Mixed venous oxygen saturation (SvO_2_)	62%
Arterial oxygen saturation (SaO_2_)	87%
Cardiac output (CO)	4.3 L/min
Cardiac index (CI)	2.5 L/min/m^2^
Pulmonary vascular resistance (PVR)	6.51WU
Pulmonary vasoreactivity testing	Negative response

The patient’s typical symptoms, along with elevated RF and CCP levels, strongly support the diagnosis of RA. The ACR/EULAR score of 8 confirms the diagnosis, while the DAS28 score of 5.68 indicates high disease activity. Treatment was initiated with oral hydroxychloroquine 0.2 g and iguratimod 25 mg twice a day, along with subcutaneous adalimumab 40 mg twice a month. Considering the comprehensive laboratory tests and examinations, the patient was diagnosed as PH secondary to RA, cardiac function class II (WHO-FC), and classified into the intermediate-low risk group. The patient received oral targeted medicine for PH macitentan 10 mg and tadalafil 20 mg once a day, besides oral diuretics of furosemide and spironolactone.After discharge, we followed up with the patient for 4 months. Within a month, we observed a significant decrease in RF, ACPA, CRP and NT-proBNP (Table 1). A subsequent chest CT indicated the remission of pneumonia (Fig. 2C and D). After 4 months, the patient underwent a systemic review. The echocardiogram displayed no obvious abnormality in the structure and function of the heart at rest (Table 2). Laboratory tests such as ESR, RF, ACPA and NT-pro-BNP all exhibited a declining trend (Table 1). The 6MWD results showed an increase to 280 meters.

## 3. Discussion

RA is an autoimmune disease primarily characterized by erosive joint inflammation, with the main pathological feature being synovitis that leads to destruction of articular cartilage and bone.^[1]^ Unlike other autoimmune diseases such as SLE, RA rarely affects blood vessels, causing rheumatoid vasculitis (RV). RV is one of the most severe extra-articular manifestations of RA, with high titers of RF and anti-CCP antibodies often present. The prevalence of RV is low, accounting for 1% to 2% of RA cases, and the mortality rate within 5 years after diagnosis can reach 40% to 50%. Skin lesions and vasoneurological involvement are the most common manifestations of RV, while pulmonary vascular involvement is less frequent (Olivé, 2020).^[8]^

When vasculitis caused by RA involves pulmonary vessels, it can lead to the development of PH. The main pathogenic mechanisms include: First, the autoimmune process plays a role in the onset of PH: Immune complexes such as RF deposit on the vessel walls, triggering immune responses and the release of local vasoactive mediators, leading to pulmonary vasculitis, thickening of the vascular intima, vasoconstriction, narrowing of the lumen, increased vascular resistance, and ultimately PH. Second, damage and dysfunction of pulmonary vascular endothelial cells cause pulmonary vascular remodeling: In patients with RA, endothelial cell damage increases the synthesis or release of endothelin, while the synthesis of vascular relaxation factor NO from endothelial sources decreases, promoting smooth muscle cell division and proliferation, and causing vasoconstriction. Pulmonary vascular wall remodeling results in PH.^[9,10]^

Precapillary PH develops in the setting of pulmonary vascular diseases is classified as PAH. PAH associated with CTDs (CTD-PAH) is the second most common subtype of PAH, accounting for 15 to 30% of all PAH patients.^[9]^ CTD-PAH usually has a particularly poor prognosis, progressing rapidly with a less favorable response to treatment, which may refer to an underlying inflammatory vasculopathy. The combination of immunosuppressive therapy with standard vasodilator therapy was proposed.^[11]^ Systemic sclerosis (SSc) and systemic lupus erythematosus (SLE) are the major causes of CTD-PAH.^[12]^ RA combined with PAH is a rare condition in clinical practice,^[13]^ and the relationship between RA and PAH has not been elucidated. Multiple observational studies have shown that the pulmonary artery systolic pressure (PASP) of RA patients detected by echocardiography higher than the healthy matched controls. There was also a higher incidence of PASP over normal threshold of 30 mm Hg.^[11]^ The occurrence of PAH was closely related to the duration of RA and the age of patients.^[14]^ A case-control study showed that patients with RA exhibited a notable decrease in right ventricular function and worse ventricular-pulmonary arterial coupling, as assessed by TAPSE and the ratio of TAPSE to right ventricular systolic pressure.^[15]^ A retrospective cohort study from Canada indicated that RA-PAH patients have an older age of onset, lower baseline mPAP, and comparable survival than idiopathic PAH patients.^[16]^

**Figure 4.**
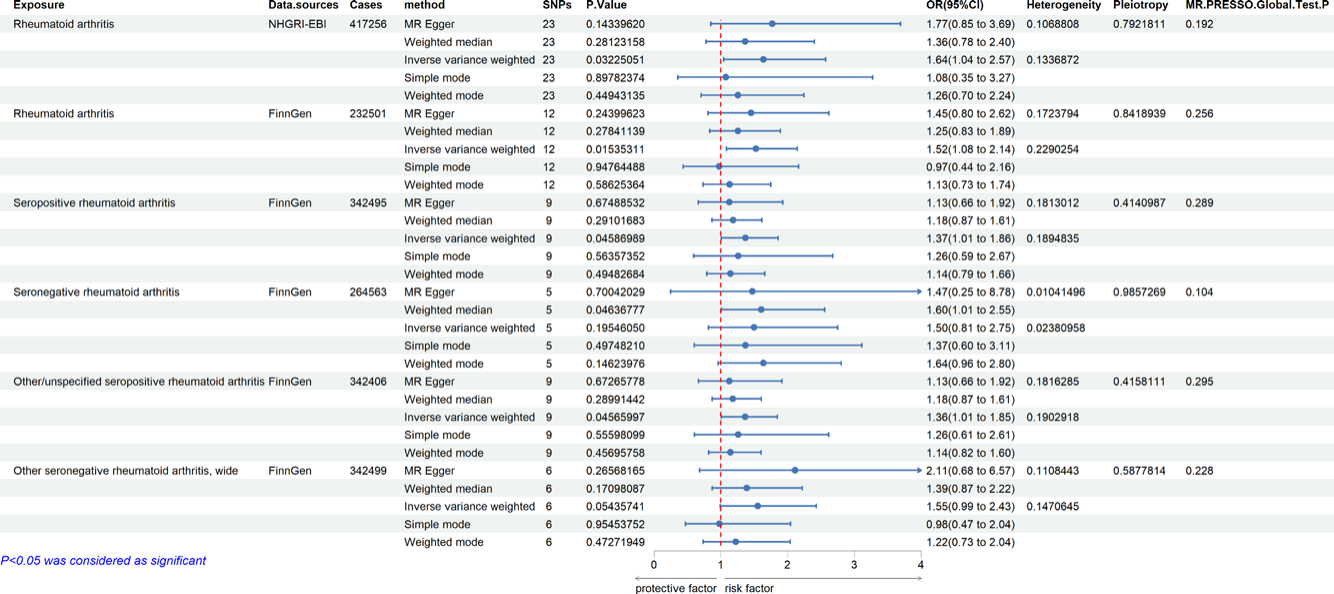
Two sample MR results: associations of RA with PAH. Results were obtained from the multiplicative random-effects inverse-variance weighted, the simple median, the weighted median, MR-Egger regression, and MR-pleiotropy residual sum and outlier (MR-PRESSO) method. The results of heterogeneity test and pleiotropy test were also shown in this figure. The MR-PRESSO analysis detected no outliers. CI = confidence interval, MR = Mendelian randomization, MR-PRESSO = MR-pleiotropy residual sum and outlier, OR = odds ratio, PAH = pulmonary artery hypertension, RA = rheumatoid arthritis, SNPs = single-nucleotide polymorphisms.

To further explore the association between RA and PAH, we designed a 2-sample MR study. This MR study was based on 3 principal assumptions. First, the instrumental variables were strongly associated with RA. Second, the instrumental variables were not affected by other confounding factors. Third, the genetic variants directly exert an influence on PAH only through RA rather than other pathways.^[7]^ We chose RA as the exposure factor in this study, and the related genetic data were obtained from 6 GWSA studies based on European individuals. The genetic data associated with PAH were obtained from FinnGen database. The result revealed that RA was causally associated with PAH by inverse-variance-weighted (IVW) approach, particularly in seropositive RA patients (OR > 1, Figure 4). Similar association was not found in seronegative patients. Sensitivity analyses such as the simple mode, the weighted mode, the weighted median, MR-Egger regression, and MR-pleiotropy residual sum and outlier (MR-PRESSO) were also conducted as complementary analyses. The heterogeneity test of IVW mode showed no evidence of heterogeneity. The pleiotropy test in MR-Egger regression and MR-PRESSO revealed no significant horizontal pleiotropy. The MR-PRESSO analysis detected no outlier. These complementary analyses confirmed the MR study results robust. There is a specific causal relationship between RA and PAH.

Inflammatory components are involved in the progression of CTD-PAH. The pathophysiological process of RA consists of 2 stages: proliferation of synovial membrane cells and infiltration of lymphocytes. Initially, the synovial layer cells proliferate, leading to the increase and activation of macrophage-like synoviocytes. These activated cells release various inflammatory cytokines, such as interleukin interleukin-1 (IL-1), interleukin-6 (IL-6) and tumor necrosis factor alpha (TNF-α). Subsequently, lymphocytes infiltrate into the lining of the synovial membrane and induce angiogenesis, which finally results in the destruction of cartilage and bone, representing the major characters of RA.^[2]^ The immune response triggered by RA not only results in joint damage but also impacts the structure and function of pulmonary vessels through the release of inflammatory cytokines. Chronic inflammation caused by CTDs involves low-level persistent infiltration of immune cells and elevated levels of several pro-inflammatory cytokines. Immune cells accumulation and infiltration are present around the pulmonary arteries, including T and B lymphocytes, macrophages, mast cells and dendritic cells, which can cause inflammatory responses and pulmonary vascular remodeling in PAH. This process involves the abnormal elevation of multiple cytokines. IL-1β could down-regulate adenosine cyclase and reduce the production of cAMP, which leads to the proliferation of vascular cells and contraction of blood vessels. IL-6 can promote the abnormal proliferation of pulmonary arterial endothelium. IL-6c synthesized and released by mast cells can induce B cells differentiation into plasma cells and production of autoantibodies. Autoantibodies such as RF deposited in the blood vessel walls could promote the proliferation of pulmonary vascular smooth muscle cells. These courses contribute to the pulmonary vascular remodeling of PAH.^[9]^ The pathological manifestations of PAH secondary to RA are consist with primary PAH, involving hypertrophy and fibroelastic proliferation of the media and the intima in small to medium-sized pulmonary arteries, inflammatory infiltrate of lymphocytes and plasma cells, and focal fibrinoid necrosis of internal and external elastic laminae.^[10]^ Therefore, RA-PAH is considered as an inflammatory vasculopathy caused by autoimmune disorders.

Modest left heart abnormalities can coexist with pulmonary vasculopathy in CTDs, manifesting as postcapillary PH, which is classified as Group 2 PH. There is a strong correlation between RA and heart failure.^[13]^ Myocardial involvement in systemic autoimmune diseases is mediated by macrovascular disease, microvascular dysfunction, and myocarditis. For RA patients, the incident of coronary heart disease, ischemic cardiomyopathy, and heart failure are elevated, and the risk of heart failure increases with disease severity.^[17]^

Various interstitial lung diseases (ILD) may also occur in CTDs, which may further progress to Group 3 PH. Scleroderma, inflammatory myopathies, and RA have the highest association with ILDs. For RA, usual interstitial pneumonia, nonspecific interstitial pneumonia, bronchiectasis and obliterative bronchiolitis are the most common histological type.^[18,19]^ The coexistence of ILDs and hypoxia may lead to a faster progression of CTDs.^[11]^

Multiple factors could contribute to the progression of RA-PH. For such patients with unexplained dyspnea, low pulse oximetry saturation, elevated NT-pro-BNP level and decreased DLCO, but without left ventricular dysfunction, RHC should be considered to confirm the diagnosis of PH.^[11]^ For this patient, although the RHC confirmed the diagnosis of CpcPH, the significant increase in mPAP and PVR, combined with echocardiography results showing normal left ventricular end-diastolic diameter and ejection fraction, suggest that the precapillary component is the dominant cause, with mild myocardial involvement. The patient’s pulmonary function tests suggested a severe decline in dispersion function characterized by a decreased DLCO and DLCO/VA. Because DLCO can be affected by lung volume, pulmonary vascular diseases usually contribute to a disproportionate reduction in DLCO with lung volume. DLCO/VA is considered as a more accurate parameter to reflect the presence of pulmonary vasculopathy.^[12]^ The patient’s pulmonary CT scan showed no evidence of significant pulmonary interstitial lesions, which proved that the decline of lung diffusion function is a consequence of extensive pulmonary vasculopathy caused by PH.

Pulmonary veno-occlusive disease (PVOD) is a particularly severe form of PAH with an extremely poor prognosis and poor response to PAH targeted therapy, which need to be identified carefully with CTD-PAH due to their similar hemodynamic features. They are both characterized by a decline of DLCO and precapillary PH. PVOD preferentially involves of small pulmonary veins together with patchy capillary proliferation, whereas PAH mainly involves the small pulmonary arteries. Chest HRCT is the best method to investigate PVOD, whose typical imaging manifestations include interlobular septal thickening, centrilobular ground-glass shadowing, and lymphadenopathy.^[12,13]^ We did not find these signatures in this patient’s pulmonary CT scan.

As for treatment, therapeutic approaches in CTD-PAH are similar to idiopathic PAH.^[6,11]^ Previous study revealed that for SSc-PAH, the combination of multiple PH-targeted drugs could improve right ventricular function and hemodynamics.^[12]^ The evidence also supports the combined application in other CTDs.^[13]^ The combination of endothelin receptor antagonists macitentan and Phosphodiesterase-5 inhibitors tadalafil was applied to the patient for the precapillary component. Macitentan could increase exercise capacity and reduce clinical worsening.^[6]^ Since the postcapillary component and left cardiac factors could not been excluded, low-dose diuretics were administered.

A variety of drugs suppressing the immune system and inflammatory responses are commonly used in the treatment of RA, such as glucocorticoids, nonsteroidal anti-inflammatory drugs, disease-modifying antirheumatic drugs (DMARDs), biological agents, and Chinese herbal medicines.^[1]^ Since the potential pathophysiologic process of CTD-PAH is due to an inflammatory vasculopathy, immunosuppressive agents and DMARDs may play a positive role in CTD-PAH.^[11]^ For SLE-PAH and MCTD-PAH, immunosuppression to control inflammation plays a more important role in the early stage of disease combining with PH-targeted therapy.^[12]^ Hydroxychloroquine is widely used in the treatment of autoimmune disease. Iguratimod as a novel DMARD can effectively down-regulate the expression of inflammation factors, and inhibit B cells producing autoantibodies. In addition, it has osteoprotective effects such as promoting bone formation and inhibiting bone destruction.^[20]^ TNF-α inhibitors and other biological agents have brought new strategies in the treatment of RA. Adalimumab, as a fully human monoclonal TNF inhibitor, has been widely used in the treatment of several immune-mediated inflammatory diseases including RA.^[21]^ The patient underwent the treatment of hydroxychloroquine, iguratimod, and adalimumab, which obtained an ideal curative effect.

## 4. Conclusion

Due to its special pathophysiological mechanism, RA can lead to different types of PH, which should be carefully identified in clinical practice. RHC is essential to assess mPAP and PVR, which is helpful for the classification of PH. Pulmonary CT scan can be used to identify patients with ILD or POVD. Pulmonary function tests can assess lung diffusion function. The combination of antirheumatic drugs and PH-targeted drugs can effectively reduce autoantibody levels and pulmonary artery pressure.

## Acknowledgments

We are most grateful to all participants in the present study.

## Author contributions

**Conceptualization:** Jingwei Liu, Weihua Zhang.

**Data curation:** Jingwei Liu.

**Formal analysis:** Jingwei Liu.

**Investigation:** Jingwei Liu, Chunyan Rong, Yin Wang, Baoguo Wang, Xuhan Liu.

**Methodology:** Baoguo Wang, Xuhan Liu, Weihua Zhang.

**Resources:** Chunyan Rong, Yin Wang, Baoguo Wang, Xuhan Liu.

**Software:** Jingwei Liu.

**Visualization:** Jingwei Liu.

**Writing – original draft:** Jingwei Liu.

**Writing – review & editing:** Weihua Zhang.
